# Combinations of Epidemiological and Experimental Studies in Air Pollution Research: A Narrative Review

**DOI:** 10.3390/ijerph17020385

**Published:** 2020-01-07

**Authors:** Hannah Weisenberg, Tianyu Zhao, Joachim Heinrich

**Affiliations:** 1 Department of Environmental Health, Rollins School of Public Health, Emory University, Atlanta, GA 30322, USA; Hannah.e.weisenberg@emory.edu; 2 Institute and Clinic for Occupational, Social and Environmental Medicine, University Hospital, Ludwig Maximilian University of Munich, 80336 Munich, Germany; 3 Comprehensive Pneumology Center (CPC) Munich, Member DZL, German Center for Lung Research, 80336 Munich, Germany; 4 Institute of Epidemiology, Helmholtz Zentrum München—German Research Center for Environmental Health, 85764 Neuherberg, Germany; 5 Allergy and Lung Health Unit, Melbourne School of Population and Global Health, The University of Melbourne, Melbourne, VIC 3010, Australia

**Keywords:** air pollution, epidemiological methods, animal experimentation, human experimentation, particulate matter

## Abstract

Scientific literature is evolving to include more systematic reviews that encompass epidemiological and experimental papers so that the whole picture can be examined. The aim of this narrative review is to bridge that gap by combining epidemiological and experimental studies based on the same setting: Examples of Bitterfeld, Utah Valley, Beijing Olympic Games, and Viadana. This review looks at four examples that incorporate multiple epidemiological and experimental papers about air pollution exposure and health effects. The Bitterfeld (spatial) and Utah Valley (temporal) examples showed that particle composition causes the biggest difference in lung injury. In Beijing, a temporal difference of before/after and during the Olympics showed that traffic and industry air pollution-related health effects like lung cancer and cardiovascular disease could be reduced by improvement of air quality. The Viadana example showed a spatial difference in respiratory injury caused by particle composition and interactions with genotoxicity. Combining experimental and epidemiological methods gives a more in-depth look into the whole picture of exposure and health effects. Our review exemplifies the strength of this strategy and encourages further use of it.

## 1. Introduction

Air pollution is one of the top causes of death globally [1]. Concern for ambient air pollution has grown over the years, especially as more countries develop and the worry for climate change grows. Nevertheless, the debate of whether air pollutants of varying levels are causing specific health effects is still ongoing. Therefore, the question of causality needs to be properly answered. Results from epidemiological studies suggest potential causal associations but fail to approve causality. Air pollution, especially particulate matter of size 2.5 micrometers or less (PM_2.5_), has been suggested as a main cause of several adverse health effects including asthma and chronic bronchitis [2]. Several epidemiological studies have reported that the size of particulate matter contributes to different health effects [3]; any organic or inorganic compounds that comprise these small particulates can then accordingly cause toxic health effects [4].

However, there is always a concern to derive a causal interpretation of epidemiological findings in current literature [5], apart from well-established Bradford-Hill criteria [6]. Despite evidence summarized by randomized controlled trials, high-quality evidence is also provided by systematic reviews of epidemiological studies in combination with experimental studies [7]. This strategy has been followed for many years by prestigious scientific institutions, such as the World Health Organization [8,9] and the Health Effects Institute [10]. Recently, some journals have published high-quality reviews [11,12,13] that combine epidemiological findings with experimental studies. However, the majority of systematic reviews are based on epidemiological studies only, or on a synthesis of different studies because of a lack of research in one setting [11,12,13,14]. Integrating results from both epidemiological and experimental examples based on the same or similar study setting could provide us a new way to synthesize and summarize the evidence. However, to the best of our knowledge, a combination of study findings from different perspectives has not been published so far in any paper. Often, studies can be spread across different journals over time, so to provide a review to present the potency of a combined approach within one setting is difficult. This narrative review aims to bridge that gap by combining epidemiological and experimental examples based on the same setting: Examples of Bitterfeld, Utah Valley, Beijing Olympic Games, and Viadana. Our review exemplifies the potency and strength of this strategy and encourages the use of it in other settings.

## 2. Materials and Methods

We conducted literature searches for examples of our interest in PubMed and selected four examples based on expert opinions for detailed presentation in this narrative review, as seen in Table 1. Based on expert opinions, we chose some well-investigated examples, two European ones—Bitterfeld and Viadana studies—one American—Utah Valley—and an example about the Beijing Olympics. These four examples were mainly arbitrarily selected because we intended not to conduct a systematic review, but to provide some views on the aforementioned combination strategy. Literature searches with a focus on particulates were conducted in the PubMed database. The relevant English examples were selected conference abstracts, and reviews were excluded. After screening the yielded hits, we included four studies on Bitterfeld, three about Utah Valley, nine about the Beijing Olympic Games, and three studies about Viadana.

## 3. Results

### 3.1. The Bitterfeld Example

#### 3.1.1. Study Design and Background

The Bitterfeld example began in the 1990s in an Eastern German region of Saxony-Anhalt [15]. The overall study looked at the impact of the local industrial pollution on the health of the local population, including but not limited to respiratory and atopic health, and how pollutants caused health effects. There were three East German cities for the study: Hettstedt and Bitterfeld, as two highly industrialized areas, and Zerbst, a rural area, which served as the control area. In Hettstedt, the main source of air pollution was mining and smelting. In Bitterfeld, the main source of air pollution was chemical processing. The different sources were hypothesized to cause different health outcomes. These areas rejoined a unified Germany in 1990. A questionnaire was sent to all the parents of children aged 5 to 14 years old in the areas. To understand the differences in asthma and allergy prevalence by town, the ambient air pollution composition was monitored. Air samples were taken between 1999 and 2000 and used in human exposure and mouse exposure experiments to see if there were different reactions to the particles from the different areas [16,17].

#### 3.1.2. Main Findings

Table 2 summarizes the main findings of the example. The paper by Heinrich et al. [15] found that asthma, allergy, and atopic dermatitis prevalence rates differed between the three regions. Bitterfeld and Hettstedt had the highest prevalence rates, which was in line with the higher amount of industrial emissions present. The children in Hettstedt also had a higher prevalence of atopic diseases compared to Zerbst children. It was speculated that this higher prevalence was caused by emissions of the copper smelter industries in Hettstedt [15].

In order to confirm this speculation, ambient particles were collected in Hettstedt and the control area Zerbst. Measurements of these particles showed higher metal contents for the Hettstedt area [16,17]. Additionally, these particles were used for human exposure studies and animal experiments. The human exposure study by Schaumann et al. [16] found that Hettstedt ambient particles instilled in human lungs caused a larger and more severe inflammatory effect and indicated that transition metals play a role in in vivo toxicity of ambient particles. Particulate matter (PM) composition may thus contribute more to respiratory illness and allergy than the amount of PM_2.5_. Gavett et al. [17] published a paper on mouse models conducted using the particle samples from Hettstedt and Zerbst. It was found that ambient PM_2.5_ from Hettstedt, which had high levels of toxic and transition metals, caused a significant increase in the number of parameters of allergic lung disease in mice, relative to PM_2.5_ from Zerbst with lower metal levels. Immunoglobulin-G levels increased after mice were sensitized to air particles from Hettstedt. These two results show that metal composition of PM_2.5_ influences allergic airway responses in subjects previously exposed to that specific PM_2.5_. A second mouse model was examined, supplementary to the Gavett paper, by Gilmour et al. [18] where PM_2.5_ particles were extracted from a filter collected in 1999 and instilled intratracheally into mice. The mice were then monitored for allergic lung disease changes and pulmonary toxicity. The particulate matter from Hettstedt had a higher mass of the metal ions zinc, lead, copper, titanium, and sodium compared to Zerbst. The mice instilled with Hettstedt particles had higher levels of inflammatory cells, worse lung injury, and edema and pro-inflammatory cytokines. Furthermore, the paper found that previous exposure to particulate matter before the allergen challenge changed allergic asthma pathophysiologic responses. This means that not only does particulate matter composition cause negative health effects but being chronically exposed can make allergy responses worse. Increased inflammatory response via cytokines can lead to chronic obstructive pulmonary disease (COPD), pneumonia, acute respiratory distress syndrome (ARDS) [19], and pulmonary fibrosis [20]. This corresponds to the 1999 paper that found a high prevalence of asthma in the children of Hettstedt [15]. This adds to the three papers in Table 2 because of the specific metal ions found to cause worse lung injury.

Biologically, the air particulate matter composition caused differing lung injuries in human exposure subjects. Though Bitterfeld and Hettstedt have marginally different air pollution levels, the Hettstedt pro-inflammatory response was different and slightly more severe than the Bitterfeld pro-inflammatory response. This was also seen in the mouse experiment when comparing sensitized mice. Mice who were not sensitized to PM_2.5_ from any region did not have an inflammatory response to the particles, whereas mice that were sensitized had an allergic airway response.

#### 3.1.3. Summary

Including all papers allows us to see the whole picture. The mouse and human exposure studies gave insight into why Hettstedt had higher rates of asthma, allergic rhinitis, allergic sensitization, and atopic dermatitis than Zerbst and Bitterfeld, though their pollution levels differed only slightly. Specifically, the experimental studies showed that the epidemiological findings of higher prevalence of atopic diseases might have been caused by higher content of transition metals in Hettstedt particles and that the epidemiologically observed regional differences were caused by variations in the metal composition of particles. This shows that the chronic allergic and asthmatic responses of children who have lived in the polluted areas for a long time were correlated with the particles present in the air.

### 3.2. Utah Valley Example

#### 3.2.1. Study Design and Background

Utah Valley in central Utah had air pollution particulate matter of 10 micrometers or less (PM_10_), monitored since 1985, and a low number of smokers. The study on human health effects of air pollution in Utah Valley area was conducted from 1985 to 1990 to assess hospital admission rates during winters with high inversion [21]. Air pollution and particulate matter monitoring were collected to determine the months that exceeded the U.S. Environmental Protection Agency (EPA) standard at the time for PM_10_ of a 24-h mean of 150 µg/m^3^, not to be exceeded more than once a year. The primary source of particulate matter was a local steel mill, which briefly closed during the study period due to a workers’ strike. PM_10_ was collected on filters from January to March 1986, 1987, and 1988. PM_10_ samples were collected in different periods of the year. Hospital admission rates for respiratory illness were collected from 1985 to 1988 from four hospitals. Monthly admissions of inpatients with diagnoses of simple pneumonia and pleurisy or bronchitis and asthma were included [21]. School attendance data for the years 1985–1986 through 1990–1991 were collected from the Provo School District and the Northridge Elementary School [22].

#### 3.2.2. Main Findings

An epidemiological study by Pope [21] found that PM_10_ levels in winters when the steel mill was open were double the amount of PM_10_ levels in winters when the steel mill was closed for an employee strike. During the months with exceedance of the 24-h PM_10_ standard, children under 18 years old had triple the hospital admissions than in months that did not exceed. When the steel mill was open in winter, admissions for children were three times as high as when the mill was closed in winter. The fall months also saw double the child admissions for bronchitis and asthma when the steel mill was open, though the 24-h mean was never exceeded.

The Ransom and Pope [22] epidemiological study also noted an association between childhood school absences and PM_10_ levels. Absences increased during periods of high PM_10_ pollution and were observed across all grade levels. The winter when the steel mill was closed had lower PM_10_ levels and lower absenteeism associated with high PM_10_ episodes.

A human exposure study by Ghio and Devlin [23] was conducted using Utah Valley air particles. PM_10_ was extracted from the filters and instilled in saline into nonsmoking human subjects. They found that the percentage of neutrophils increased after instillation of 1986 and 1988 particulate matter, as well as greater oxidative stress. There was an elevation of lung inflammation and injury after instillation of extracts from when the steel mill was open.

Utah Valley was a unique study, as the health effects of a closed steel mill versus an open one could be studied. The steel mill was noted to emit 82% of all industrial sources of PM_10_ [21]. It closed in August of 1986 during a labor dispute and reopened in September of 1987, allowing PM_10_ and the health effects of PM_10_ to be evaluated. PM_10_ levels in the winter of 1986/87 never exceeded the EPA 24-h mean standard [24]; however, the winter before (1985/86) and after (1987/88), saw PM_10_ levels higher than the EPA 24-h mean standard by 13 and 10 times, respectively. The winter months with the steel mill open had nearly three times as many hospital admissions as when the steel mill was closed. During the fall months, when there was no 24-h mean standard exceedance, but the steel mill was open, bronchitis and asthma hospital admissions for children were twice as high as those for the same months when the steel mill was closed. It can be concluded that the steel mill was correlated not only to the concentration of air pollution, but the composition as well, which in turn affected the respiratory health of the children in the valley.

#### 3.2.3. Summary

These studies show that not only PM_10_ is a threat to the health of children and adults [21,23], but the particle composition of the pollution leads to an increased inflammatory response and oxidative stress. When the steel mill was open, the studies identified much higher levels of hospital admission and school absences.

### 3.3. Beijing Olympic Games Example

#### 3.3.1. Study Design and Background

Beijing is well-known for suffering a very high concentration of air pollution, brought about by the high number of automobiles that continue to increase, the rapid development and industry in the area, and the stable weather and wind direction [25]. Before the Olympics in 2008, there was concern for the health and safety of athletes due to inhalation of air particles [26]. Beijing carried out several actions to reduce the air pollution for the Olympic Games. The following studies are focused on this special time window to investigate the variations in air pollution and health effects.

A 2016 study [27] collected weekly PM_2.5_ samples from two sites. The samples were chemically analyzed, and meteorological data were obtained. From January 2007 to December 2008, medical records were collected from one hospital in Beijing looking for emergency visits (ER visits) classified as cardiovascular diseases, hypertension, ischemic heart disease, cardiac arrhythmia, congestive heart failure, and cerebrovascular disease [28]. Meteorological data and data on sulfur dioxide (SO_2_), nitrogen dioxide (NO_2_), PM_2.5_, and PM_10_ were also collected during the same time period from monitoring sites. Another study looked at birth weight after the Olympics, with certain months of gestation falling during the event [29]

Shang et al. [30] explained how they collected PM_2.5_ samples before and during the 2008 Olympics and chemically analyzed them. They then cultured the particles extracted and performed a quantitative real-time polymerase chain reaction. A cohort of 119 subjects from a central Beijing hospital was enrolled, and electrocardiographic measurements and blood samples were taken for plasma analysis [31]. Air pollution and weather were measured and analyzed from monitors on the roof of the same hospital campus.

#### 3.3.2. Main Findings

The epidemiological study by Chen et al. [27] using air samples found that there was less pollution on days with the highest traffic regulations. They recommended continuing some of the restrictions in order for pollution levels to diminish in the urban area. Another study in 2016 [28] found an increase in cardiovascular ER visits associated with an increase in PM_2.5_, with stronger associations found in arrhythmia and cerebrovascular disease ER visits. There was also a reduction in cardiovascular ER visits associated with a reduction in air pollution during the Olympics. The 2015 study [29] found that babies whose eighth months of gestation were during the Olympics had a significant increase in birth weight, averaging 23 grams larger than babies born in 2007 or 2009 with the same calendar month for the eighth-month gestation period. They concluded that even short-term decreases in air pollution would have had an effect on birth weight.

The Shang et al. [30] study found that pollution restrictions during and before the Beijing Olympics caused a decrease in certain chemical components in air pollution and thus a lower cytotoxic response. Several compounds collected during the 2008 Olympics were found to have toxic cellular responses, such as zinc, causing macrophage cell death and no cytokine release, among others. Rich et al. [31] found a decline in inflammatory and cardiovascular disturbance biomarkers when air pollution in Beijing decreased during the Olympics. Risk of lung cancer also reduced during the Olympics [32]. A 2014 study in Beijing from 2007 to 2011 collected PM_2.5_ samples on filters from two sites. They were chemically analyzed and evaluated for element differentials [33]. Elements with enrichment factors (EFs) greater than 50 are generally known to be caused by anthropogenic sources. The air samples analyzed had seven compounds with EFs greater than 50, including copper, tin, zinc, lead, arsenic, cadmium, and antimony.

#### 3.3.3. Summary

The results of all these papers not only show that air pollution is detrimental to health, but also that different pollutants have different effects, as seen in Beijing around the time of the 2008 Olympic Games. The Shang et al. [30] study showed the biological basis for what the epidemiological studies observed on low traffic days around the time of the Beijing Olympics. The Rich and Jia papers also highlight other aspects of health affected by the decrease in pollution during the time of the Olympics. They support each other to enhance the results and suggest that Beijing’s high traffic and industry, a single setting, cause the high concentration of air pollution, as well as a multitude of accompanying health effects like lung cancer and cardiovascular disease.

### 3.4. Viadana Example

#### 3.4.1. Study Design and Background

The Viadana study in Italy created many papers that were experimental and epidemiological in nature. They focused on a pediatric population that lived near two chipboard factories in Northern Italy from 1996 to 2005. These factories created wood dust and air particles with chemical compounds. A questionnaire was sent out in 2006 to inquire about local children’s health and respiratory symptoms [34]. A second questionnaire was sent out in 2010–2011 (Viadana II) to a random sample of participants from the first survey [35]. Children from the second survey had mouth cells collected. The cells were then assayed and processed. Air pollution was monitored in the region for NO_2_ (an intermediate in the oxidation of nitrogen oxides) and formaldehyde.

#### 3.4.2. Main Findings

Generally, gaseous air pollution is of more concern in this area than particles. An epidemiological paper by de Marco et al. [34] saw an impact on health status, most notably eye symptoms and cough/phlegm, in the children living less than 2 km from the chipboard factories. Symptoms, school absences, and emergency department visits or hospitalizations were also found to decrease with increasing distance from the source. A 2011 epidemiological paper based on the de Marco study [36] saw an increase in hospital admissions for respiratory diseases and symptoms as children lived closer to the chipboard factories, with those living within 2 km of the factories having the highest admission rates.

The paper by Marcon et al. [35] used experimental studies to find that formaldehyde, often used with wood processing, and NO_2_ were causing genotoxic shifts in the children being studied. Children living less than 2 km from the chipboard factories had higher formaldehyde and NO_2_ genotoxic shifts than the other children. Mucosal exposure was higher in children living closer to the factories. Spatially, formaldehyde and NO_2_ in the Viadana area were more concentrated around the chipboard industrial areas. The children who had more exposure to these compounds and pollutants also had higher levels of genotoxic shift markers.

#### 3.4.3. Summary

Together, these three papers show that the chipboard factory was detrimental to the health of the children living nearby. The chemical exposure-related children’s respiratory symptoms were caused by genotoxic shifts and cell damage [34,35,36].

## 4. Discussion

Our review synthesized both epidemiological and experimental examples for four typical study clusters: Bitterfeld, Utah Valley, Beijing Olympic Games, and Viadana. The epidemiological studies investigated air pollution-related health effects and were cross-referenced by the experimental studies in these four cases. More specifically, the higher prevalence of atopic diseases might be caused by a higher content of heavy metals in particulate matter in Bitterfeld [15,16,17,18]. The studies in Utah Valley indicated that inflammatory response and oxidative stress might be involved in the particle-related child admissions for bronchitis and asthma, and school absences [21,22,23]. The studies in Beijing covered a broad spectrum of health effects and provided a more comprehensive insight into associations between particles’ components and diseases [25,26,27,28,29,30,31,32,33]. Finally, the Viadana studies indicated that the chemical exposure-related respiratory symptoms were caused by genotoxic shifts and cell damage [34,35,36]. We can, therefore, see that without experimental studies, observed health effects and correlations cannot be backed up with biological evidence and in-depth knowledge to strengthen the argument.

Based on the characteristic of exposure in these studies, the Bitterfeld [15,16,17,18] and Viadana [34,35,36] examples were spatial contrasts, as they showed the regional differences in exposure. The Utah Valley [21,22,23] and Beijing [25,26,27,28,29,30,31,32,33] examples were temporal contrasts due to the differences in particle levels and health effects by time. One strength of our review is that we summarized evidence to answer foundational questions regarding two study designs: Regional (spatial) and time-variant (temporal). However, for regional comparisons, because several other unmeasured factors could be responsible, the reasons regarding the observed regional differences are always questionable. For the time-variant studies, several other factors that correlate with time variation might have caused the time-dependent effects and leave the identified associations in question. Thus, additional experimental studies are extremely beneficial in backing up the epidemiological findings with biological causality evidence to eliminate concerns arising from the limitations of simple spatial or temporal contrast designs. This can be seen in Table 2, which lays out the findings and comparisons of each study. It shows how the studies compare against each other as well.

These examples are different to other systematic reviews because each example is based on a single setting. A single setting is best for conducting well-rounded studies to ensure comprehensive evidence. Without the human effect study, we would not be able to see how the different air samples cause differing levels of lung injury in the Bitterfeld example. Without the epidemiological study, we would not know that air pollution fluctuates with the mill operation and corresponds with fluctuating allergy and asthma trends in the Utah Valley example. To the best of our knowledge, this is the only known review that combines all types of studies conducted on a similar population, in the same area, over the same amount of time. Usually, reviews adopt data from a variety of studies with heterogenous air particles. A study by Lim et al. [37] on particulate matter and children’s hospital admissions, for example, used 26 different studies from North America and Europe, and each study had a different sample.

Another strength of single-setting studies is that these types of studies are also good temporally, as the researchers can work simultaneously, in spite of their different professions, and without interference. The Bitterfeld [15,16,17,18] example is especially strong because the air samples taken from the regions being studied were used in both the human exposure and mice studies.

Although we would like to encourage these types of studies and reviews, the vast cost input would be an obstacle for researchers to carry out such far-ranging studies. These types of reviews can also be difficult and complicated due to the level of access to samples across multiple collaborators. It can be challenging to find all the key articles necessary for the bigger picture.

As a narrative review trying to synthesize evidence, we did not conduct a systematic search of studies. We likely missed some research about the four examples, as well as further research in additional scenarios, due to the use of solely the PubMed database and inclusion of English language studies only. Another limitation would be the arbitrarily selected four examples, which were mainly on the basis of previous knowledge. Therefore, we may have neglected some other possible examples that could be summarized. However, we conducted this narrative review with the intent of providing a view of the combination strategy, to summarize the strength of single-setting studies, and to encourage the use of this strategy in future air pollution research.

## 5. Conclusions

The review synthesized epidemiological and experimental studies for four typical study clusters. Each of the above examples take place in a specific area and within a specific time window, allowing the possibility for researchers to conduct different studies among a similar population with similar exposure, to thus gain more comprehensive evidence on exposure to air pollution and health effects. We advocate adopting this same-setting strategy to combine pollution-related epidemiological and experimental evidence in future studies.

## Figures and Tables

**Table 1:** A list of the searches in the PubMed database (up to 25 October 2019) completed for the examples presented in this paper.

Study	Search	Hits	Relevant Hits
Bitterfeld	(((“Bitterfeld”[tiab]) AND (pollutant *[tiab] OR pollution[tiab] OR pm[tiab] OR pm2.5[tiab] OR pm10[tiab] OR “particulate matter *”[tiab] OR “particle *”[tiab])) AND English[Language])	24	4 *
Utah Valley	(((“Utah valley”[tiab]) AND (pollutant *[tiab] OR pollution[tiab] OR pm[tiab] OR pm2.5[tiab] OR pm10[tiab] OR “particulate matter *”[tiab] OR “particle *”[tiab])) AND English[Language])	27	21
Beijing Olympic Games	(((“Beijing”[tiab] OR “Peking”[tiab]) AND “olympic”[tiab]) AND (pollutant *[tiab] OR pollution[tiab] OR pm[tiab] OR pm2.5[tiab] OR pm10[tiab] OR “particulate matter *”[tiab] OR “particle *”[tiab])) AND English[Language])	72	50
Viadana	(((“Viadana”[tiab]) AND (pollutant *[tiab] OR pollution[tiab] OR pm[tiab] OR pm2.5[tiab] OR pm10[tiab] OR “particulate matter *”[tiab] OR “particle *”[tiab])) AND English[Language])	5	5

* One paper was manually added.

**Table 2:** Comparison of single-setting studies. This table compares the four studies (Bitterfeld, Utah Valley, Beijing, and Viadana) to show the pros and cons of each.

Type	Example	Location and Time	Design	Population	Environmental Pollution				Epidemiology						Human Exposure	Animal Model
					PM	Heavy Metals	NO_x_	Formaldehyde	Asthma	Hay Fever	Lung Function	Atopic Dermatitis	Lung Injury from Inflammation			
Spatial	Bitterfeld	Germany (1992–2004)	Hettstedt compared to Zerbst	Children	↑	↑	NA	NA	↑	↑	*↑	↑	NA		Increase in inflammatory response to Hettstedt samples	Increase in inflammatory response to Hettstedt samples
	Viadana	Italy (1996–2015)	<2 km from factory compared to >2 km from factory	Children	NA	NA	↑	↑	↑	↑	NA	NA	↑		More likely to have genotoxic shifts in cells of children	NA ***
Temporal									**Asthma**	**Cardiovascular Health**	**Lung Injury from Inflammation**	**Bronchitis**	**School Absences**	**Hospital Admissions**		
	Utah Valley	Utah, USA (1985–1991)	Winter with Steel Mill Open compared to Steel Mill Closed	Children, adults (experimental)	↑	↑	NA	NA	↑	NA	NA	↑	↑	↑	High number of neutrophils and inflammation	NA ***
	Beijing	Beijing (2007–2016)	Before Compared to During Olympics	Adults	↓	↓	↓	NA	NA	↑	↓	NA	NA	NA	** toxic cellular responses	NA ***
		Beijing (2007–2016)	During Olympics Compared to After	Pregnant Women, Adults	↑	↑	↑	NA	NA	↑	↑	NA	NA	NA	lower cytotoxic responses	

* Over time as air pollution and air particle concentration decreased; ** from air samples collected during the Olympics; *** NA refers to the fact that these types of papers were not found during our search in PubMed before December 2019; **↑**/**↓** refers to the increase/decrease (respectively) in the column title as mentioned in the paper.
